# 2425. A Cost-effectiveness Analysis of Chlorhexidine Bathing and Nasal Decolonization in General Medical and Surgical Units: Comparing Universal and Targeted Strategies

**DOI:** 10.1093/ofid/ofad500.2044

**Published:** 2023-11-27

**Authors:** Lyndon P James, Natasha Stout, Taliser R Avery, Sarah Stein, Kenneth E Sands, Julia Moody, Eunice J Blanchard, Russell Poland, Richard Platt, Susan Huang

**Affiliations:** Harvard University, Boston, Massachusetts; Harvard Medical School, Boston, Massachusetts; Harvard Pilgrim Healthcare Institute, Boston, Massachusetts; Harvard Pilgrim Health Care Institute, Boston, Massachusetts; HCA Healthcare, Nashville, Tennessee; HCA Healthcare, Nashville, Tennessee; HCA Healthcare, Nashville, Tennessee; HCA Healthcare, Nashville, Tennessee; Harvard Pilgrim Health Care Institute, Boston, Massachusetts; University of California, Irvine School of Medicine, Irvine, CA

## Abstract

**Background:**

Hospital-associated bloodstream infections (BSI) are a major source of morbidity and healthcare costs. The ABATE (Active BAThing to Eliminate infection) trial found that universal chlorhexidine bathing (plus nasal mupirocin for MRSA carriers) in general medical and surgical units significantly reduced BSI among those with medical devices (central lines, midlines and lumbar drains), but not necessarily among other non-ICU patients. Whether to implement targeted decolonization among those with devices (TD) vs universal decolonization (UD) depends on the balance between health benefits and costs.

**Methods:**

We constructed a decision analytic model to simulate BSI frequency and costs under three strategies: No decolonization (ND), TD, and UD. In TD, decolonization was given only to those with medical devices and adherence was varied in sensitivity analyses. We performed the analysis from the perspectives of the payor and of individual hospitals. Product costs were included under both perspectives. For payors, the costs of BSI events were included. For hospitals, while BSI costs were not accrued in the base case, we explored the value in reducing BSI by varying the hospital’s willingness-to-pay (WTP) to avoid one BSI. Model parameters were informed by the ABATE trial and additional literature, and we explored the sensitivity of results to parameter variations.

**Results:**

In the base case, TD was dominant (i.e., cost-saving). Compared to ND, TD prevented 0.64 BSI and saved $16,551 per 1,000 admissions under the payor perspective. From the hospital perspective, UD may be preferred in settings with greater proportions of patients with devices, higher WTP to avert BSI, larger reductions in BSI among those with devices, and/or lower adherence to targeted bathing (Figure).

Four-way deterministic sensitivity analysis from the hospital perspective

**Figure:**
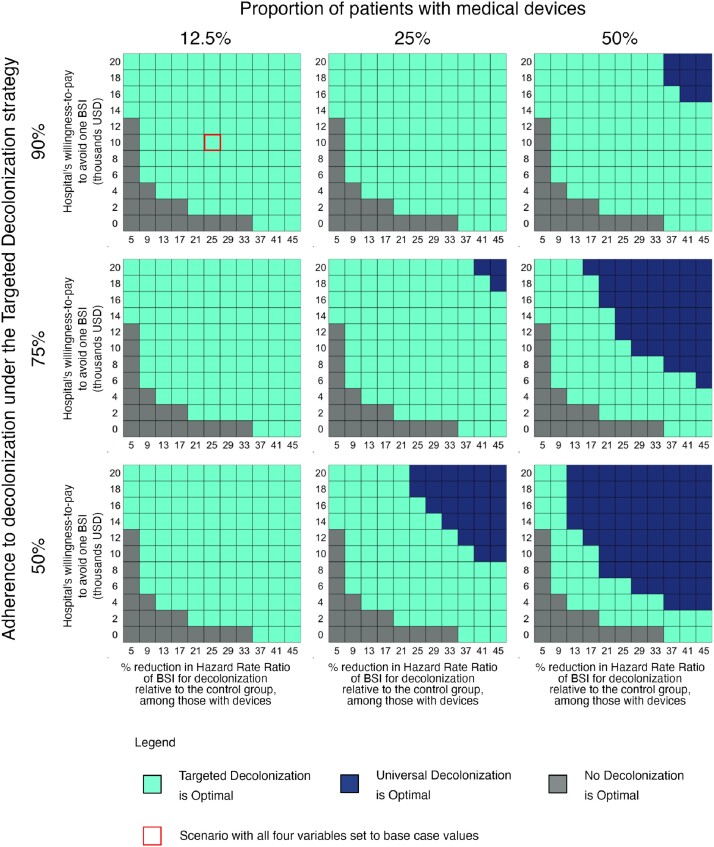

The optimal strategy is shown as a function of the proportion of patients with devices (12.5%, 25%, 50%), the adherence to decolonization under the TD strategy (50%, 75%, 90%), the treatment effect for decolonization among those with devices (5% to 45% reduction in the post vs. pre implementation HRR of BSI as compared to the standard of care), and the hospital’s willingness-to-pay to avoid one BSI. All other variables are held at the base case values for the hospital perspective. The optimal strategy is the one that provides the best value for money, given the hospital’s specified willingness-to-pay to avoid BSI. BSI – bloodstream infection; HRR – hazard rate ratio; USD – United States dollars

**Conclusion:**

TD reduces BSI and cost under a broad range of scenarios for hospitals and healthcare payors. Further reductions in BSI achievable from UD – which come at additional expense – may be merited when at least two of the following are true: device use (or prevalence of other high risk conditions) is high, financial penalties associated with BSI are high, decolonization is better than the trial point estimate for those with devices, or adherence to decolonization is greater for UD vs TD due to work flow adoption issues.

**Disclosures:**

**Susan Huang MD Huang, MD, MPH**, Medline: Conducting studies whereby participating nursing homes and hospital patients receive contributed antiseptic or environmental cleaning products|Xttrium: Conducting studies whereby participating nursing homes and hospital patients receive contributed antiseptic product

